# Growing a New Study: Environmental Influences on Child Health Outcomes

**DOI:** 10.1289/ehp.123-A260

**Published:** 2015-10-01

**Authors:** Charles W. Schmidt

**Affiliations:** Charles W. Schmidt, MS, an award-winning science writer from Portland, ME, has written for *Discover Magazine*, *Science*, and *Nature Medicin*e.

Prevalence rates for asthma, autism spectrum disorders, obesity, attention-deficit/hyperactivity disorder, and many other chronic childhood diseases remain stubbornly high in the United States.^1^ It is also now widely understood that environmental exposures in early development and even preconception can adversely affect an individual’s health long after childhood.^2^ Environmental factors in child development therefore affect the health not just of children themselves but of all society. Now the National Institutes of Health (NIH) is developing a large-scale long-term program to better understand these factors, dubbed Environmental Influences on Child Health Outcomes (ECHO).

**Figure:**
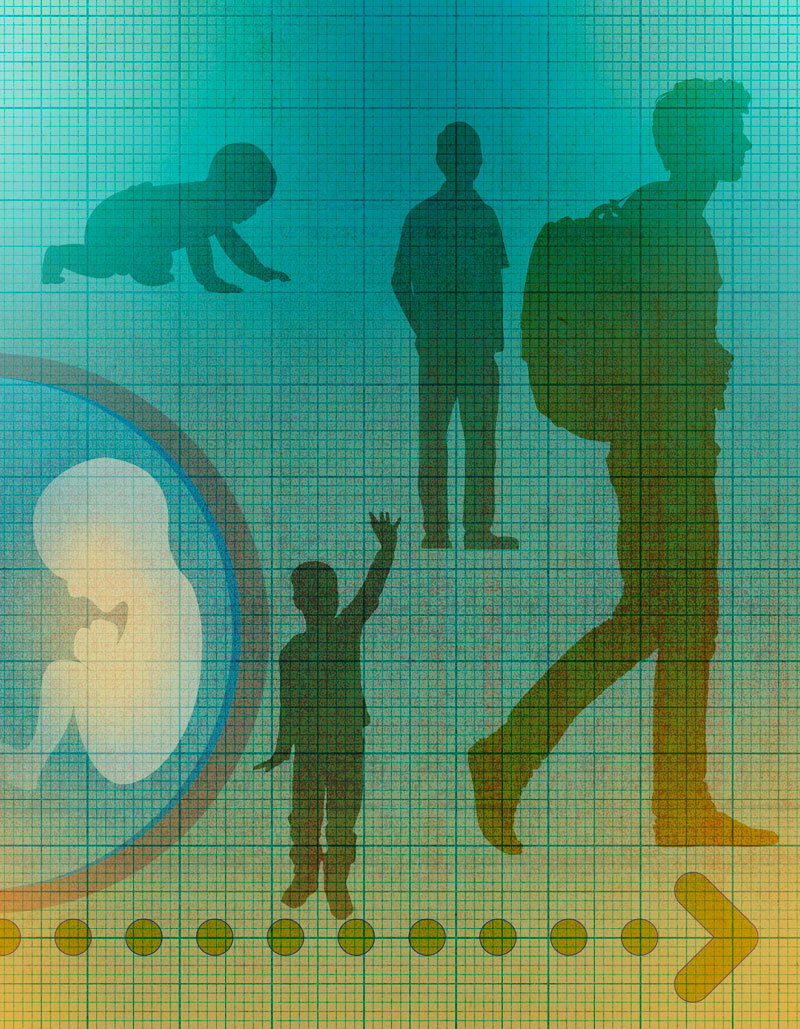
A new program known as Environmental Influences on Child Health Outcomes will take advantage of existing birth cohorts, and possibly new cohorts, as well to study key areas of concern to children’s development and health. © Roy Scott

NIH leaders envision building ECHO around four areas of public health interest: 1) upper- and lower-airway conditions, such as asthma and allergies; 2) obesity and related conditions such as diabetes and metabolic syndrome; 3) pre-, peri-, and postnatal outcomes, including birth defects; and 4) neurodevelopment and related conditions and outcomes such as autism, behavior, and cognition. Standardized core elements expected to be measured across each focus area will include demographics, growth, sleep, nutrition, and activity patterns, among others, plus newer parameters enabled by more recent scientific advances, including those pertaining to the microbiome and epigenetic influences on childhood development.

In a Request for Information (RFI) issued 13 July 2015, the NIH solicited input on these plans.^3^ Approximately 190 comments were received. As a general theme, the comments applauded the NIH’s effort to pull together a large, racially diverse study, while also raising concerns about how ECHO will integrate data sets derived from different sources and address the effects of early-life exposures, especially during gestation.

## Building on the National Children’s Study

In a first foray into such broad-scale investigation of children’s environmental health, Congress in 2000 directed the NIH to carry out a prospective birth-cohort study that would follow 100,000 U.S. children from pregnancy to at least 21 years of age. In the National Children’s Study (NCS) researchers planned to measure chemical exposures in pregnancy and during early postnatal life, and to bank biological and environmental samples for later analysis. By 2014 more than $1.3 billion had been allocated to the NCS.^4^ But NIH director Francis Collins cancelled the study in December of that year^5^ after a review by the Institute of Medicine and recommendations from the Advisory Council to the Director concluded that the NCS was plagued by design flaws and feasibility issues.^6^

Lawrence Tabak, deputy director of the NIH, emphasizes that ECHO won’t be just a new NCS, however. “We’re maintaining programmatic goals,” he says, “but the approach is totally different.”

A critical difference between the NCS and ECHO concerns the use of birth cohorts. Where the NCS set out to enroll a large new cohort, ECHO will rely on existing cohorts and repositories of tissues—such as cord blood and placenta—that have been collected to measure environmental exposures and epigenetic changes across pregnancy and during childhood. “Making full use of available infrastructure by drawing on existing cohorts means we don’t have to reinvent the wheel and go through this costly process of recruitment,” Tabak says. “Existing cohorts will have done that already.”

According to Dean Baker, a professor and director of the Center for Occupational and Environmental Health at the University of California, Irvine, the plan to recruit a new birth cohort was part of NCS’s downfall. Baker, a former investigator of a NCS study center who later served on the Institute of Medicine review committee, explains that study planners opted for household-based recruitment using a door-to-door strategy that would ideally enroll large numbers not only of pregnant women but also of women who were planning to become pregnant. This would enable the evaluation of environmental effects from the earliest stages of development onward.

Field staff achieved initial recruitment goals during the NCS pilot phase. But follow-up of the nonpregnant women proved more problematic. “During the pilot, we were limited to traditional telephone communication, and the preconception population is highly mobile—many young women move and change phone numbers frequently,” Baker explains. He says investigators planned to follow the women for five years but had lost contact with 54% of them within a year and a half.

Brenda Eskenazi, director of the Center for Environmental Research and Children’s Health at the University of California, Berkeley, points out that hospital or clinic-based recruitment improves on door-to-door sampling “because you’re working with women who are already in the medical system.” And indeed, based on additional pilot testing, the NCS program office did eventually propose a more practical strategy of provider-based and hospital prenatal recruitment specifically for pregnant women. “But by that time,” Baker says, “Congress had mandated the review of the NCS by the Institute of Medicine, which concluded in its 2014 report^6^ that the NIH program responsible for NCS lacked sufficient in-house expertise and the NCS management structure was unlikely to produce a high-quality, cost-efficient study protocol.”

## Existing Cohorts, New Tools

NIH officials have not yet selected any existing cohorts for ECHO research funding. But Tabak has made public reference^7^ to the Nulliparous Pregnancy Outcomes Study: Monitoring Mothers-to-Be—or nuMoM2b for short—a cohort of racially, ethnically, and geographically diverse pregnant women recruited at eight clinical research sites and twelve subsites around the country.^8^ Launched in 2010 by the Eunice Kennedy Shriver National Institute of Child Health and Human Development (NICHD) and co-funded by NICHD and the NIH Office of Research on Women’s Health, nuMoM2b has recruited 9,000 women so far and ultimately aims to recruit 10,000 in all. The cohort was designed to characterize the genetic, epigenetic, and environmental factors that predict adverse pregnancy outcomes among mothers and developing babies. NICHD principal investigators who direct the cohort did not respond to requests for comment.

But several researchers interviewed for this story are concerned that many existing birth cohorts in the United States are irrelevant in some respect to the environmental exposures children face today. Eskenazi, for instance, directs the CHAMACOS (Center for the Health Assessment of Mothers and Children of Salinas) Study, which investigates environmental factors and child health in a population of Latino agricultural workers in California’s Salinas Valley.^9^ The study enrolled 601 pregnant women at six local clinics between 1999 and 2000. Some data collected years ago when the study was launched might still be valuable, Eskenazi says, but they may not reflect current environmental exposures, such as alternative chemicals used to replace known toxicants.

Baker also questions how many existing cohorts collected high-quality data on environmental conditions, especially during critical periods of prenatal development. “That really isn’t clear,” he says, “although I think it’s a good idea to inventory what’s out there to see what we can merge together.”

According to Baker, most of the prospective studies of environmental influences on child health during the last decade have been statistically underpowered—that is, limited by having too few participants for certain types of analysis. Therefore, he says, the total population drawn from existing cohorts in ECHO would need to be large enough to assess multiple exposures—and interactions between exposures and genes—with statistical confidence.

Nigel Paneth is a professor of epidemiology, biostatistics, and pediatrics at Michigan State University and a former NCS principal investigator. He emphasizes that to identify preventable risk factors for childhood disease, especially the dominant causes of infant mortality—birth defects and preterm birth^10^—researchers must measure environmental exposures comprehensively in real time during pregnancy. “ECHO, by restricting itself to extant cohorts, will provide some information, but it’s not comparable to making a comprehensive assessment during the critical exposure window of pregnancy,” he says.

Philip Landrigan, former director of the Children’s Environmental Health Center at New York’s Mount Sinai Hospital, recommends a hybrid approach: the judicious creation of new birth cohorts and/or the recruitment of new mother–infant pairs into selected existing cohorts. “This strategy will allow investigators to assess new environmental exposures that did not exist when some of the existing cohorts were first created a decade or more ago,” he says.

Landrigan and Baker coauthored a perspectives article in early 2015^1^ in which they argued for a “coordinated national confederation of regional, academically based, prospective birth-cohort studies” that could pursue the NCS’s initial research agenda. That confederation, they wrote, “would collect, analyze, store, and share common core data under standard protocols, but each institution could also collect data specific to its population, environment, and geographic region.” Baker says that ECHO appears to be “a meaningful alternative to the NCS,” but he argues the program will need a longer-term strategy for pooling and analyzing data from existing cohorts, adding new births to those cohorts, and initiating new birth cohorts.

One of ECHO’s expected strong points will be that it can serve as a test for new tools used in environmental and pediatric monitoring. “The NCS didn’t make the investments needed for developing sensors and other types of sophisticated exposure technology,” Baker says. “We’re talking about chips that can measure a huge array of chemicals at very low concentrations. That might sounds like science fiction, but people are working on these tools, and they’re making progress.”

The National Institute of Environmental Health Sciences is supporting the development of many such sensors, says institute director Linda Birnbaum. “We provided leadership in the NIH Roadmap Epigenetics Program^11^ and have continued this support through our regular grants programs,” she says. Indeed, the NIH just awarded nearly $144 million in new grants to develop new tools and approaches for studying pediatric diseases, which will help support ECHO.^12^

## Next Steps

A long-term strategy for ECHO has not been decided, in part because it will depend on funding availability. “We anticipate five to seven years of funding and are planning with this timeline in mind,” Tabak says. According to a statement provided to *EHP* by the NIH press office, the President’s budget requests $165 million in support for ECHO for fiscal year 2016.

The NIH is now reviewing the comments submitted in response to the RFI, and an analysis will be prepared and released in the coming months. Summaries are provided to the ECHO working group on a regular and ongoing basis, Tabak says, and key items will continue to be discussed and considered for implementation into the final ECHO plan. Tabak anticipates that fiscal year 2016 funding opportunity announcements will be released later this year, with the review process beginning next summer. Awards are expected to be made by September 2016.
